# Development of methodological approaches to conducting training to reduce occupational stress based on the mindfulness method among medical personnel of psychiatric institutions

**DOI:** 10.1192/j.eurpsy.2026.11713

**Published:** 2026-07-31

**Authors:** V. Mitikhin, L. Alieva, T. Solokhina

**Affiliations:** FSBSI “Mental Health Research Center”, Moscow, Russian Federation

## Abstract

**Introduction:**

The presence of occupational stress among medical workers in psychiatric facilities is a factor that can negatively affect not only their quality of life and well-being, but also the quality of medical care provided to patients (Solokhina T. et al., 2024). Considering the results obtained (Oshevsky D. et al., 2025), it is relevant to develop and implement technologies aimed at stress prevention and psychological support for medical personnel. One of the approaches to the prevention of occupational stress may be the Mindfulness-based stress reduction (MBSR) method proposed by J. Kabat-Zinn (1990-2013). This approach has been successfully applied when dealing with anxiety, depressive, and psychosomatic disorders (Tomasino B. et al., 2016; Fujino et al., 2018; Zollars I. et al., 2019). The main principles of MBSR are to develop the skills of acceptance, openness, lack of self-esteem, letting go, trust, patience, and self-compassion.

**Objectives:**

To develop methodological approaches to conducting training to reduce occupational stress based on MBSR among medical personnel of psychiatric institutions.

**Methods:**

25 nurses from psychiatric institutions in Moscow participated in the pilot study. During the development of the training, MBSR techniques were used, as well as author’s developments (Alieva L. et al., 2016).

**Results:**

The training consisted of eight weekly sessions, the objectives of which were to develop the skills of constructive response to stressful situations, psychophysical self-regulation, and attention management. The solution to these tasks was provided by informing about stress, conducting discussions, and a set of special exercises developed by us. The meeting lasted two hours. Each lesson included an informational part and practical approaches, which included a set of exercises and tasks aimed at developing awareness of thoughts, emotions, and bodily sensations. To adapt the technology we developed based on MBSR, participants received homework assignments aimed at consolidating their acquired skills. At the end of the training, nurses improved their awareness of the connection between the cognitive, emotional, and bodily components of self-perception, expanded their repertoire of self-regulation skills, and increased their level of self-awareness.

**Conclusions:**

The developed training to reduce occupational stress using mindfulness techniques has demonstrated its promising outlook. It is necessary to further implement it and evaluate its effectiveness. In the future, it can be recommended as a short-term method for the prevention of occupational stress among the medical staff of psychiatric institutions.

**Disclosure of Interest:**

None Declared

